# Association between sleep duration and hearing threshold shifts of adults in the United States: National Health and Nutrition Examination Survey, 2015–2016

**DOI:** 10.1186/s12889-023-17204-3

**Published:** 2023-11-21

**Authors:** Lili Long, Yuedi Tang

**Affiliations:** 1 Department of Otorhinolaryngology, Sichuan University Hospital of Sichuan University, Chengdu, Sichuan China; 2 Department of Otorhinolaryngology Head and Neck Surgery, West China Hospital of Sichuan University, No. 37 Guo-Xue-Xiang, Chengdu, 610041 Sichuan China

**Keywords:** Sleep duration, Hearing threshold, Adults, Cross-sectional study, National Health and Nutrition Examination Survey

## Abstract

**Background:**

Obstructive sleep apnea (OSA) is linked to hearing loss (HL). Another sleep characteristics, sleep duration might also be associated with HL, but prior evidence is limited. This study is aimed to investigate the association between sleep duration and hearing level in the adult US population.

**Methods:**

In total, a sample of 2777 individuals aged 20–69 years from the 2015–2016 National Health and Nutrition Examination Survey cycle (NHANES, 2015–2016) were investigated in this study. Self-reported sleep duration data was classified into the short-sleep (< 7 h), normal-sleep (7–9 h), and long-sleep (> 9 h) group. Multivariable linear regression models between sleep duration and hearing threshold shifts were estimated. Interactions between sleep duration and age, gender, race, OSA were also considered, and the study population was stratified by age, gender, race, and OSA to analyze the potential disparities among adults in different subgroups.

**Results:**

Long-sleep duration was positively associated with speech- and high-frequency pure-tone average (PTA) thresholds with statistical significance (β = 1.31, 95%CI: 0.10, 2.53, *P* = 0.0347, and β = 2.71, 95%CI: 0.69, 4.74, *P* = 0.0087, respectively). When stratified by age, short sleep duration was positively associated with low-, and speech-frequency PTAs (*P* = 0.0140 and 0.0225, respectively) for adults aged 40–59 years, and long-sleep duration was positively associated with low-, and speech-frequency PTAs (*P* = 0.0495 and 0.0142, respectively) for adults aged 60–69 years with statistical significance. There was statistically significant interaction between OSA and sleep duration on speech-frequency PTA, but no significant interaction between either gender or race with sleep duration on hearing thresholds among US adults.

**Conclusion:**

Short/long sleep durations are associated with worse hearing level comparing to sleep 7–9 h in the American adults. Nonoptimal sleep duration may be a potential risk factor for HL.

**Supplementary Information:**

The online version contains supplementary material available at 10.1186/s12889-023-17204-3.

## Background

Hearing loss (HL) is a highly prevalent form of sensory deficit and global public health problem. It creates a heavy social and financial burden, and reduces people’s quality of life by the loss of education, limited communication and social interaction which accompanies unaddressed HL [1]. Furthermore, HL is the largest modifiable risk factor for dementia and is associated with poorer physical functioning and mortality among elders [2–4]. Globally, over 1.5 billion people of all ages experience some degree of HL currently, and the number of people with HL could grow to 2.5 billion by 2050 according to the World Health Organization [1]. Understanding of the factors contributing to HL is of great importance.

Sleep disorders are of high prevalence. One study reported that 35.7% of Chinese residents aged 15–69 years old had poor sleep quality, according to the China Chronic Disease and Risk Factor Surveillance Study 2007 [5]. Millions of Americans suffer from chronic sleep disorders [6]. Sleep disorders include insomnia, circadian rhythm abnormality, obstructive sleep apnea (OSA), poor sleep quality, and nonoptimal sleep duration. Plausible potential mechanisms (e.g., disturbed energy metabolism, disrupted cochlear blood flow and increased oxidative stress) may link sleep disorders with HL [7–15]. However, there is limited evidence regarding the relationship between sleep disorders and HL, and most research has focused on exploring the association between OSA and HL [16–20]. Previous Research has shown that people with OSA have worse hearing at higher frequencies [21].

Another sleep disorder is sleep duration. One third of the American adults reported shorter sleep duration than the amount recommended by the National Sleep Foundation [22]. Nonoptimal sleep durations have been correlated with adverse outcomes. Too short sleep duration has been associated with hypertension, cardiovascular disease, obesity, and type II diabetes, whereas too long sleep duration has been linked with cardiovascular disease, stroke, and multiple sclerosis [23]. Sleep duration might also be associated with HL. One of the main structures of the peripheral auditory system is the cochlea, the sensory hair cells in which are highly vulnerable to the reactive oxygen species (ROS). ROS accumulate during wakefulness, and antioxidants remove excess ROS during sleep. Inadequate sleep can be harmful to the process of removing ROS. The plausible mechanism prompts the relationship between sleep duration and HL. However, only four cross-sectional studies have investigated the association in adults, based on Japanese, American, Chinese and the UK population [24–27], the results of which are inconsistent.

Here, we performed a cross-sectional study investigating the association between sleep duration and hearing level in adults aged 20–69 who participated in the National Health and Nutrition Examination Survey (NHANES) 2015–2016 in the US, which is the most recent survey cycle including both sleep duration data and audiometric-exam data performed among adults between 20–69 years old, to provide more evidence to test the hypothesis that adults with abnormal sleep duration are more likely to have worse hearing.

## Methods

### Study design and population

NHANES is a continuous nationwide series of surveys that collect health-related cross-sectional representative data of the US general population on a 2-year basis. NHANES program consists of in-home interviews, physical examinations and laboratory tests of a selected sample of the noninstitutionalized US population using a complex, multistage probability sampling design. This study analyzed publicly available data downloaded from the NHANES official website.

**Fig. 1:**
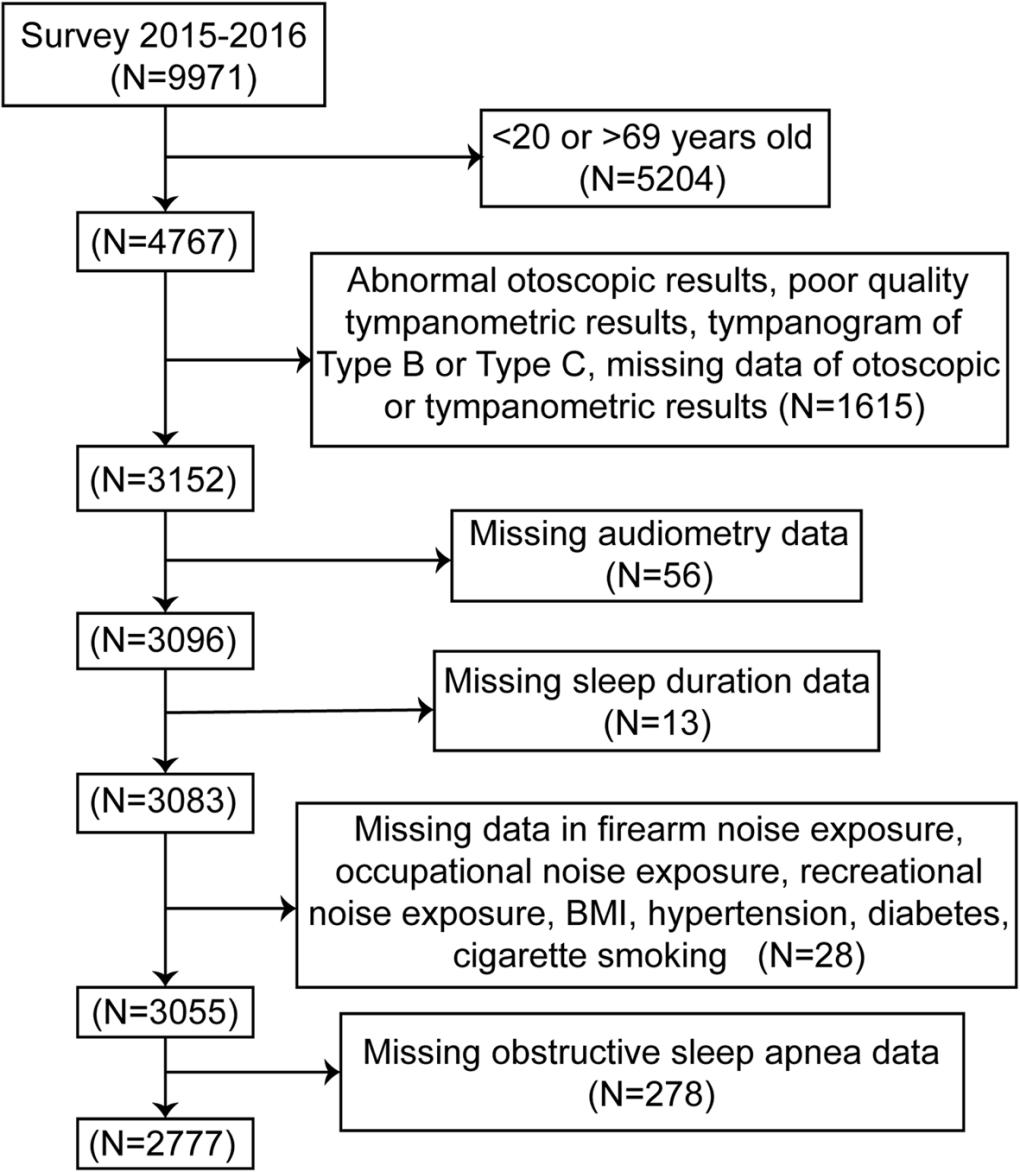
Flow chart of the selection process (only participants aged 20–69 years were eligible for audiometric testing in the 2015–2016 NHANES survey cycle). NHANES, National Health and Nutrition Examination Survey

The participants in this study were enrolled from the NHANES cycle 2015–2016, which contains results of both sleep duration data and audiometric-exam data of 20–69 years old adults. Figure 1 shows the flow chart of participant selection in this study. The participants with abnormal otoscopic results, poor-quality tympanogram results or tympanogram of Type B or Type C were excluded to avoid analyzing conductive or mixed HL data [28–30]. Individuals with missing information of hearing threshold levels, otoscopic test, tympanogram test, firearm noise exposure, occupational noise exposure, recreational noise exposure, body mass index (BMI), hypertension, diabetes, cigarette smoking were also excluded (*n* = 28). Participants with missing OSA data were excluded (*n* = 278). Finally, 2777 Individuals were included in our study.

### Sleep duration assessment

In this study, sleep duration information was obtained from the NHANES Sleep Disorders Questionnaire, which was publicly available on the website (https://wwwn.cdc.gov/Nchs/Nhanes/2015-2016/SLQ_I.htm). The reported sleep duration to question “How much sleep usually get at night on weekdays or workdays?” was rounded to the nearest half-hour. The short-sleep group was defined as individuals with self-reported sleep duration less than 7 h, the long-sleep group contained participants with sleep duration more than 9 h, and the normal-sleep group included participants sleeping 7–9 h [31].

### Audiometric measurement

Audiometric measurement was conducted in the mobile examination center by trained examiners. Participants aged 20–69 years were eligible. The tympanometry was examined using the Titan tympanometer. The pure tone air conduction audiometry of both ears was measured with an Interacoustics Model AD226 audiometer, TDH-49P headphones and Etymotic EarTone 3A insert earphones. Participants unable to remove hearing aids or could not tolerate headphones were excluded. The full procedure was described in detail on the website (https://wwwn.cdc.gov/Nchs/Nhanes/2015-2016/AUX_I.htm).

In this study, low-frequency pure-tone average (PTA) was defined as PTA hearing thresholds of 0.5, 1, and 2 kHz in the better ear, speech-frequency PTA was defined as PTA hearing thresholds of 0.5, 1, 2 and 4 kHz in the better ear, and high-frequency PTA was defined as PTA hearing thresholds of 4, 6, and 8 kHz in the better ear. HL was defined as speech-frequency PTA ≥ 20 dB in the better ear, according to the suggestion of WHO (2021) [1].

### Potential covariates

Potential covariates in the report included: age, gender, race, education level, BMI, hypertension, diabetes, cigarette smoking, firearm noise exposure, occupational noise exposure, recreational noise exposure, and OSA. Information on age, gender, race, education level, hypertension, diabetes, cigarette smoking, firearm noise exposure, occupational noise exposure, recreational noise exposure, and OSA was obtained during in-home interviews. BMI data were obtained during physical examination.

Self-reported hypertension was assessed by an affirmative answer to either the question “Have you ever been told by a doctor or other health professional that you had hypertension, also called high blood pressure?” or the question “Because of your high blood pressure/hypertension, have you ever been told to take prescribed medicine?” Hypertension measured was the mean blood pressure value of three measurements ≥ 130 mm Hg for systolic blood pressure or ≥ 80 mm Hg for diastolic blood pressure [32]. Participants were considered diabetes if they met one of the following conditions: fasting plasma glucose ≥ 126 mg/dL, glycated hemoglobin ≥ 6.5%, oral glucose tolerance test ≥ 200 mg/dL, currently using insulin or oral hypoglycaemic agents, and reported diabetes or sugar diabetes on the questionnaire [33]. Smoking status was classified as never smoker, former smoker and current smoker from the questions, “Have you smoked at least 100 cigarettes in your entire life?” and “Do you now smoke cigarettes?” [34]. Firearm noise exposure was defined as “ever used firearm for any reason” [35]. Occupational noise exposure was defined as “ever had a job exposure to loud noise for 4 or more hours a day, several days a week” [35]. Recreational noise exposure was defined as “outside of a job, ever been exposed to very loud noise or music for 10 or more hours a week” [35]. OSA was defined based on answers to three questions, including: (1) snoring 3 or more nights per week; (2) snorting, gasping, or stopping breathing 3 or more nights per week; or (3) feeling excessively sleepy during the day 16–30 times per month despite sleeping around 7 or more hours per night on weekdays or work nights. Individuals positive for symptoms of OSA answered yes to at least one of the preceding 3 questions [36].

### Statistical analysis

A *P*-value of less than 0.05 was considered of statistical significance. All analyses were performed using R 3.6.1 and R 4.3.1 with continuous data presented as mean ± standard deviation and categorical data shown as percentages (N%). Full sample 2 year interview weight (WTINT2YR) of the 2015–2016 cycle was applied to adjust for over-sampling, survey nonresponse, noncoverage, and poststratification, as suggested, which allows it to represent the civilian noninstitutionalized US population [37, 38]. Weighted descriptive statistics characterizing the samples in demographic and potential hearing-related confounders among participants were estimated. To analyze the distribution of covariates across the sleep duration categories, *P*-values of continuous variables and categorical variables were calculated by weighted linear regression model and weighted chi-square test, respectively.

The relationship between sleep duration and hearing threshold shifts were assessed using multivariable linear regression models. Both the unadjusted linear regression model (crude model) and the model adjusted for confounders were applied, using the normal-sleep group as the reference group. Regression coefficients (β) and 95% confidence intervals (CIs) were determined in the linear regression models. The adjusted model as adjusted for age, gender, race, education level, BMI, hypertension, diabetes, cigarette smoking, firearm noise exposure, occupational noise exposure, recreational noise exposure, and OSA.

The interactions of sleep duration with age, gender, race, and OSA were analyzed to test whether the relationship between sleep duration and hearing level differed by age, gender, race or OSA. *P* values for interaction (*P*_interaction_) were calculated by including a cross-product term in the multivariable linear regression models. *P*_interaction_ < 0.05 was considered evidence of a possible interaction. Subgroup analyses of sleep duration stratified by age, gender, race, and OSA were performed. Participants were classified as 20–39 years age group, 40–59 years age group, and 60–69 years age group based on the suggestion of the Center for Disease Control and Prevention (https://wwwn.cdc.gov/nchs/nhanes/tutorials/module3.aspx).

## Results

### Baseline characteristics of study participants

Table 1 shows that participants in the study (n = 2777) aged between 20 and 69 years (weighted mean, 42.55 ± 13.86 years) included weighted 55.23% females and weighted 44.77% males. The rate of HL was 16.97%, 19.02%, and 12.39% in normal-, short-, and long-sleep group, respectively. Compared with participants in the normal-sleep group, participants in the short-sleep group were significantly older, had higher prevalence of HL, slightly higher BMI (mean 30.30 vs 29.17 kg/m^2^), more likely to be male (55.56% vs 43.68%), more likely to be Black (18.80% vs 8.17%), less educated (above high school: 62.56% vs 72.87%). They were also significantly more likely to be smoker (46.92% vs 37.86%), more likely to have hypertension (50.12% vs 38.84%) and diabetes (15.23% vs 12.63%), more likely to expose to firearm or occupational noise (53.47% vs 50.98%, 38.71% vs 29.69%, respectively), and more likely to have OSA (54.10% vs 46.30%). Compared with participants in the normal-sleep group, individuals in the long-sleep group were significantly younger, had lower prevalence of HL, slightly lower BMI (mean 29.28 vs 29.17 kg/m^2^), more likely to be Black (15.23% vs 8.17%), less likely to be male (30.22% vs 43.68%), less likely to expose to firearm noise (39.54% vs 50.98%) and less educated (above high school: 48.51% vs 72.87%), but more likely to have hypertension (42.41% vs 38.84%) and diabetes (18.38% vs 12.63%). They were slightly more likely to be smoker (41.53% vs 37.86%) with statistical significance. There were no statistical differences among different sleep duration groups in low-, speech-, and high-frequency hearing thresholds and proportion of adults exposed to recreational noise (all *P* > 0.05).

**Table 1:** The weighted demographic characteristics of study participants by sleep duration. (NHANES 2015–2016 cycle, *N* = 2777)

Variables	Total	Normal-sleep (*N* = 1864)	Short-sleep (*N* = 622)	Long-sleep (*N* = 291)	*P*^a^
Age, years	42.55 ± 13.86	43.00 ± 13.85	43.11 ± 12.81	37.42 ± 15.08	** < 0.0001**
Low-frequency PTA, dB^b^	9.65 ± 9.07	9.47 ± 8.61	10.45 ± 11.06	9.48 ± 7.78	0.0876
Speech-frequency PTA, dB^b^	11.71 ± 10.29	11.57 ± 9.89	12.61 ± 12.21	10.91 ± 8.73	0.0563
High-frequency PTA, dB^b^	22.65 ± 18.76	22.70 ± 18.53	23.60 ± 20.39	20.00 ± 16.50	0.0504
BMI, kg/m^2^	29.39 ± 7.24	29.17 ± 7.09	30.30 ± 7.60	29.28 ± 7.55	**0.0066**
Male, %	44.77	43.68	55.56	30.22	** < 0.0001**
Race, %					** < 0.0001**
Mexican American	9.33	8.59	11.33	11.32	
Non-Hispanic White	63.46	67.77	51.21	53.15	
Non-Hispanic Black	10.74	8.17	18.80	15.23	
Other races	16.48	15.48	18.66	20.31	
Education level, %					** < 0.0001**
Below high school	11.78	10.01	15.14	19.73	
High school	19.31	17.12	22.30	31.76	
Above high school	68.91	72.87	62.56	48.51	
Hypertension, %	41.24	38.84	50.12	42.41	** < 0.0001**
Diabetes, %	13.59	12.63	15.23	18.38	**0.0260**
Cigarette smoking, %					** < 0.0001**
Never smoker	60.14	62.13	53.08	58.47	
Former smoker	22.06	23.35	21.97	10.98	
Current smoker	17.80	14.51	24.95	30.55	
Firearm noise, %	50.49	50.98	53.47	39.54	**0.0014**
Occupational noise, %	31.47	29.69	38.71	30.83	**0.0004**
Recreational noise, %	15.12	14.69	17.95	12.59	0.0963
OSA, %	47.59	46.30	54.10	44.32	**0.0038**
^c^Hearing loss, %	16.97	16.97	19.02	12.39	0.0824

*Abbreviations*: *PTA* pure-tone average, *BMI* body mass index, *OSA* obstructive sleep apnea

^a^*P* values of continuous variables and categorical variables were calculated by weighted linear regression model and weighted chi-square test, respectively

^b^Low-, speech-, high-frequency PTA values in the better ear were computed from the average of hearing thresholds of 0.5, 1 and 2 kHz, 0.5, 1, 2 and 4 kHz, 4, 6 and 8 kHz, respectively

^c^Hearing loss was defined as the PTA value ≥ 20 dB in the better ear

### Multivariable regression analysis: association of sleep duration with hearing threshold shifts

Multivariable linear regression analyses were performed using the normal-sleep group as the reference group (Table 2). In the crude model, when comparing to the normal-sleep group, long sleep duration and high-frequency PTA hearing threshold shifts in the better ear exhibited a statistically significant inverse correlation (β = -2.70, 95% CI: -5.25, -0.16, *P* = 0.0376), while the hearing thresholds of the short-sleep group were positively related to both low-, and speech-frequency PTAs with statistical significance (β = 0.98, 95% CI: 0.10, 1.85, *P* = 0.0290, and β = 1.04, 95% CI: 0.05, 2.03, *P* = 0.0396, respectively). After adjusting for age, gender, race, education level, BMI, hypertension, diabetes, cigarette smoking, firearm noise exposure, occupational noise exposure, recreational noise exposure, and OSA, long sleep duration was positively correlated with both higher speech-, and high-frequency PTAs with statistical significance (β = 1.31, 95% CI: 0.10, 2.53, *P* = 0.0347, and β = 2.71, 95% CI: 0.69, 4.74,* P* = 0.0087, respectively), while no significant difference was observed in the short-sleep group.

**Table 2:** Multivariable linear regression models assessing the relationship between sleep duration and PTA hearing thresholds

Sleep duration	Crude Model			Adjusted Model		
**Low-frequency PTA**	β	95% CI	*P*	β	95% CI	*P*
Normal-sleep	Ref			Ref		
Short-sleep	0.98	0.10, 1.85	**0.0290**	0.65	-0.17, 1.46	0.1201
Long-sleep	0.00	-1.23, 1.24	0.9959	1.02	-0.13, 2.18	0.0826
**Speech-frequency PTA**						
Normal-sleep	Ref			Ref		
Short-sleep	1.04	0.05, 2.03	**0.0396**	0.43	-0.43, 1.29	0.3306
Long-sleep	-0.66	-2.05, 0.74	0.3565	1.31	0.10, 2.53	**0.0347**
**High-frequency PTA**						
Normal-sleep	Ref			Ref		
Short-sleep	0.90	-0.91, 2.71	0.3310	0.07	-1.36, 1.50	0.9245
Long-sleep	-2.70	-5.25, -0.16	**0.0376**	2.71	0.69, 4.74	0.0087

Crude Model = unadjusted. Adjusted Model = Crude Model + age, gender, race, education level, BMI, hypertension, diabetes, cigarette smoking, firearm noise exposure, occupational noise exposure, recreational noise exposure, OSA. *PTA* pure tone average, *CI* confidence interval, *normal-sleep* sleep duration between 7 and 9 h

### Subgroup analyses

Statistically significant interactions between sleep duration and age were found on the association between sleep duration and both low-, and speech-frequency PTAs (*P*_interaction_ = 0.0108 and *P*_interaction_ = 0.0064, respectively), but not on the association between sleep duration and high-frequency PTA (*P*_interaction_ = 0.0962) (Table 3). Statistically significant interaction between sleep duration and OSA was found on the association between sleep duration and speech-frequency PTA (*P*_interaction_ = 0.0445) (Supplementary Tables S1). No statistically significant interactions of sleep duration with gender or race were found (all *P*_interaction_ > 0.05) (Supplementary Tables S2 and S3). In the subgroup analyses stratified by age, short sleep duration was positively correlated with low-, and speech-frequency PTAs with statistical significance (β = 1.37, 95% CI: 0.28, 2.47, *P* = 0.0140, and β = 1.20, 95% CI: 0.17, 2.23, *P* = 0.0225, respectively) for adults 40–59 years of age, and long sleep duration was positively correlated with low-, and speech-frequency PTAs with statistical significance (β = 2.99, 95% CI: 0.01, 5.96, *P* = 0.0495, and β = 3.65, 95% CI: 0.74, 6.55, *P* = 0.0142, respectively) for adults 60–69 years of age, when controlling for age, gender, race, education level, BMI, hypertension, diabetes, cigarette smoking, firearm noise exposure, occupational noise exposure, recreational noise exposure, and OSA (Table 3). In the subgroup analyses stratified by OSA, both short-, and long-sleep durations were positively correlated with speech-frequency PTA with statistical significance (β = 1.07, 95% CI: 0.12, 2.02, *P* = 0.0282, and β = 1.36, 95% CI: 0.10, 2.61, *P* = 0.0344, respectively) for adults without OSA, when controlling for age, gender, race, education level, BMI, hypertension, diabetes, cigarette smoking, firearm noise exposure, occupational noise exposure, and recreational noise exposure (Supplementary Tables S1).

**Table 3:** Adjusted^a^ associations between sleep duration and PTA hearing thresholds stratified by age (*N* = 2777)

Age, years	Normal-sleep	Short-sleep		Long-sleep		*P*_interaction_
		β (95% CI)	*P*	β (95% CI)	*P*	
**Low-frequency PTA**						**0.0108**
≥ 20, < 40 (*N* = 1261)	Ref	0.15 (-0.66, 0.96)	0.7165	0.90 (-0.07, 1.86)	0.0679	
≥ 40, < 60 (*N* = 1092)	Ref	1.37 (0.28, 2.47)	**0.0140**	-0.61 (-2.57, 1.35)	0.5416	
≥ 60, < 69 (*N* = 424)	Ref	-0.44 (-2.38, 1.51)	0.6597	2.99 (0.01, 5.96)	**0.0495**	
**Speech-frequency PTA**						**0.0064**
≥ 20, < 40 (*N* = 1261)	Ref	-0.18 (-0.97, 0.61)	0.6527	0.91 (-0.03, 1.85)	0.0570	
≥ 40, < 60 (*N* = 1092)	Ref	1.20 (0.17, 2.23)	**0.0225**	-0.29 (-2.14, 1.55)	0.7561	
≥ 60, < 69 (*N* = 424)	Ref	-0.15 (-2.05, 1.75)	0.8784	3.65 (0.74, 6.55)	**0.0142**	
**High-frequency PTA**						0.0962
≥ 20, < 40 (*N* = 1261)	Ref	-1.55 (-2.89, -0.20)	**0.0244**	0.27 (-1.33, 1.87)	0.7385	
≥ 40, < 60 (*N* = 1092)	Ref	1.41 (-0.41, 3.24)	0.1283	2.23 (-1.03, 5.50)	0.1798	
≥ 60, < 69 (*N* = 424)	Ref	3.00 (-1.45, 7.44)	0.1867	-4.00 (-8.15, 0.15)	**0.0293**	

^a^Adjusted for age, gender, race, education level, BMI, hypertension, diabetes, cigarette smoking, firearm noise exposure, occupational noise exposure, recreational noise exposure, and OSA

## Discussion

In this nation-wide cross-sectional study, we explored the relationship between sleep duration and hearing threshold shifts among adults aged 20–69 years using the NHANES 2015–2016 database. Our study found that compared with normal sleep duration, long sleep duration was statistically significantly associated with worse speech-, and high-frequency hearing, after adjusting for confounders. Statistically significant interactions between sleep duration and age were found on the associations between sleep duration and both low-, and speech-frequency PTAs. When stratified by age, short sleep duration (< 7 h of sleep) was statistically significantly associated with worse low-, and speech-frequency PTAs for adults aged 40–59 years, and long sleep duration (> 9 h of sleep) was statistically significantly associated with poorer low-, and speech-frequency PTAs for adults aged 60–69 years, compared with normal sleep duration. Statistically significant interaction between sleep duration and OSA was found on the association between sleep duration and speech-frequency PTA. When stratified by OSA, both short-, and long-sleep were statistically significantly associated with poorer speech-frequency PTA for adults without OSA, compared with normal sleep duration. There was no statistically significant interaction between either gender or race with sleep duration on PTA hearing threshold shifts among US adults. There is an enduring need for implementation of prevention strategies to reduce the potential disease burden associated with HL.

The results of multivariable linear regression analysis in our study are broadly consistent with the three previous population-based studies among adults in both the United states and Japan, which reported association of long sleep duration (sleep duration exceeded 8 h) with worse hearing [24–26], but are inconsistent with the conclusions drawn from studies in the population from UK and China, which indicated no association or a negative association between long sleep duration and HL [26, 27]. The correlations of short sleep with worse low-, and speech-frequency hearing were found for US adults 40–59 years of age when stratified by age. This result differs from the previous studies [24–27]. A cross-sectional and longitudinal study conducted by Nakajima, K et al. investigated 48,091 and 6,674 healthy Japanese general population, respectively, illustrating a potential positive relationship between longer sleep duration and subclinical HL among adults aged 20–79 years, without adjusting noise exposure [24]. The result is the same based on the data from 2011–2012 NHANES cycle [26]. Another cross-sectional study conducted by Jiang, K et al. investigated 632 US adults aged 70 years and older using the NHANES 2005–2006 database, suggesting marginal association of longer sleep duration with poorer high-frequency hearing, detecting no difference among participants by age or race [25]. One Chinese population based cross-sectional study has reported negative relationship between sleep duration ≥ 8 h/night and HL [26]. One longitudinal study including 231,650 UK participants aged 38–72 years old has shown no relationship between sleep duration and the risk of HL [27]. Racial heterogeneity, differences in sample size, study design and available covariate data may possible explanations of the disparities between our results and the previous studies. The NHANES survey cycle 2015–2016 is the most recent survey cycle including both sleep duration data and audiometric-exam data performed among adults between 20–69 years old. The findings of our study can add to the results of the research done by Jiang, K et al. on US adults aged 70 years and older using the 2005–2006 NHANES cycle database to get a whole concept of the association between sleep duration and hearing among US adults from the young to the old.

The potential mechanism underlying the relationship between sleep duration and hearing is not clear. Though previous studies have linked long sleep duration to diseases and adverse outcomes, such as hypertension, diabetes, cognitive decline and mortality, which are closely associated with HL [7–15, 39], the physiological or biological mechanisms linking longer sleep duration and these diseases are unclear. Activities in the interleukin 6 and C-reactive protein inflammatory pathways were reported to be elevated by excessive sleep duration, suggesting that inflammatory signals may play a potential role in causing HL in people with long sleep duration [40]. The results of previous experiments suggested that inflammatory factors and defect of cochlear out hair cells may be the potential pathogenesis of HL caused by short sleep. An animal experiment indicated that sleep deprivation can induce increased proinflammatory cytokine IL-1β and decreased distortion product otoacoustic emission levels (indicator of the function of cochlear outer hair cells) and result in HL in Wistar rats [41]. IL-1β may contribute to inner ear fluid retention through affecting epithelial Na^+^ channels in the endolymphatic sac [42]. ROS may also play a role in hearing impairment in people with inadequate sleep. One of the main structures of the peripheral auditory system is the cochlea, the sensory hair cells in which are highly vulnerable to ROS. ROS accumulate during wakefulness, and antioxidants remove excess ROS during sleep. Inadequate sleep can be harmful to the process of removing ROS.

Both nonoptimal sleep duration and OSA were reported to be associated with hearing disorders in epidemiological studies [21, 24–26]. It was thought previously that nonoptimal sleep duration may influence the hearing level partly by sleep-disordered breathing caused hypoxic or ischemic conditions. However, our findings show that both short-, and long-sleep durations were positively correlated with speech-frequency PTA with statistical significance for adults without OSA, which indicating that sleep duration may have an independent effect on hearing threshold. Though the mechanism of OSA on hearing impairment is not clear, there are some evidences indicating that eustachian tube disorder and a potential decline in cochlear function may play roles in it [21]. The relationship between OSA and central auditory processing is little known.

There are some strengths and limitations in this study. The data we analyzed is from a nationally representative sample of US population, obtained from NHANES, which is large, standardized and reliable. Despite these strengths, some limitations of this study should be addressed. First, inferring temporality does not allowed for the NHANES is a cross-sectional study. Abnormal sleep duration may be a marker or a risk factor for worse hearing. Second, both the information of sleep duration and the feature of OSA were obtained from self-reported measures, which are subjective and not accurate enough. Third, only main confounders reported in previous studies based on prior knowledge were adjusted in this study, which cannot exclude the influence of all the other potential confounders, and there are some potential confounders we could have wished to control but were unable to calculate in our study, like chlorinated pollutants or proximity to dioxin sources as incinerators. Furthermore, a mechanistic exploring of the effect of abnormal sleep duration on hearing impairment is lacking. Thus, further studies are needed to confirm our findings.

## Conclusion

According to the findings of NHANES analyses, long sleep duration (> 9 h of sleep) was positively correlated with speech-, and high-frequency hearing with statistical significance for US adults. When stratified by age, short sleep duration (< 7 h of sleep) was statistically significantly associated with worse low-, and speech-frequency hearing for adults aged 40–59 years old, and long sleep duration was statistically significantly correlated with poorer low-, and speech-frequency hearing for adults aged 60–69 years old. When stratified by OSA, both short-, and long-sleep were positively correlated with speech-frequency hearing with statistical significance for adults without OSA. Both short and long sleep duration were detrimental to hearing in US adults of certain groups. Implementation of prevention strategies to reduce the potential disease burden associated with HL may be needed. Future prospective longitudinal studies with increased sample size are needed. Studies using other analysis method, like Mendelian randomization analysis may also improve our approach. Besides, basic experimental studies and functional studies will help to understand the biological mechanisms underlying the relationship between sleep duration and HL.

### Supplementary Information

**Additional file 1: Table S1.** Adjusted^a^ associations between sleep duration and PTA hearing thresholds stratified by OSA (*N *= 2777). **Table S2.** Adjusted^a^ associations between sleep duration and PTA hearing thresholds stratified by gender (*N *= 2777). **Table S3.** Adjusted^a^ associations between sleep duration and PTA hearing thresholds stratified by race (*N *= 2777).

## Data Availability

Datasets and study protocols from NHANES are publicly available via the NHANES website (https://www.cdc.gov/nchs/nhanes/Index.htm).
