# Supplementary material for: Association between sleep duration and hearing threshold shifts of adults in the United States: National Health and Nutrition Examination Survey, 2015–2016
Source: BMC Public Health. 2023 Nov 21;23:2305. doi: 10.1186/s12889-023-17204-3 (PMC10664608; doi:10.1186/s12889-023-17204-3)
Supplement: Supplementary file 1 — Additional file 1: Table S1. Adjusteda associations between sleep duration and PTA hearing thresholds stratified by OSA (N = 2777). Table S2. Adjusteda associations between sleep duration and PTA hearing thresholds stratified by gender (N = 2777). Table S3. Adjusteda associations between sleep duration and PTA hearing thresholds stratified by race (N = 2777). [file 12889_2023_17204_MOESM1_ESM.docx]

Table S1 Adjusted^a^ associations between sleep duration and PTA hearing thresholds stratified by OSA (N = 2777)

| OSA | Normal-sleep | Short-sleep | | Long-sleep | | *P*_interaction_ |
| --- | --- | --- | --- | --- | --- | --- |
|  |  | β (95% CI) | *P* | β (95% CI) | *P* |  |
| **Low-frequency PTA** |  |  |  |  |  | 0.0523 |
| No (N = 1392) | Ref | 1.31 (0.31, 2.31) | **0.0104** | 1.09 (-0.22, 2.41) | 0.1032 |  |
| Yes (N = 1385) | Ref | -0.14 (-0.96, 0.69) | 0.7456 | -0.18 (-1.43, 1.08) | 0.7825 |  |
| **Speech-frequency PTA** |  |  |  |  |  | **0.0445** |
| No (N = 1392) | Ref | 1.07 (0.12, 2.02) | **0.0282** | 1.36 (0.10, 2.61) | **0.0344** |  |
| Yes (N = 1385) | Ref | -0.40 (-1.18, 0.37) | 0.3072 | -0.07 (-1.26, 1.11) | 0.9017 |  |
| **High-frequency PTA** |  |  |  |  |  | 0.4372 |
| No (N = 1392) | Ref | 0.49 (-1.19, 2.16) | 0.5698 | 2.29 (0.08, 4.49) | **0.0422** |  |
| Yes (N = 1385) | Ref | -0.65 (-2.23, 0.94) | 0.4250 | 1.51 (-0.92, 3.93) | 0.2240 |  |

^a^ Adjusted for age, gender, race, education level, BMI, hypertension, diabetes, cigarette smoking, firearm noise exposure, occupational noise exposure and recreational noise exposure.

Table S2 Adjusted^a^ associations between sleep duration and PTA hearing thresholds stratified by gender (N = 2777)

| Gender | Normal-sleep | Short-sleep | | Long-sleep | | *P*_interaction_ |
| --- | --- | --- | --- | --- | --- | --- |
|  |  | β (95% CI) | *P* | β (95% CI) | *P* |  |
| **Low-frequency PTA** |  |  |  |  |  | 0.8226 |
| Female (N = 1539) | Ref | 0.59 (-0.39, 1.57) | 0.2392 | 0.40 (-0.77, 1.56) | 0.5036 |  |
| Male (N = 1238) | Ref | 0.63 (-0.19, 1.45) | 0.7458 | 1.50 (0.01, 2.99) | **0.0488** |  |
| **Speech-frequency PTA** |  |  |  |  |  | 0.7334 |
| Female (N = 1539) | Ref | 0.44 (-0.48, 1.37) | 0.3485 | 0.74 (-0.35, 1.84) | 0.1840 |  |
| Male (N = 1238) | Ref | 0.21 (-0.59, 1.02) | 0.6014 | 1.07 (-0.38, 2.53) | 0.1495 |  |
| **High-frequency PTA** |  |  |  |  |  | 0.0708 |
| Female (N = 1539) | Ref | 0.45 (-1.07, 1.97) | 0.5625 | 2.29 (0.49, 4.09) | **0.0126** |  |
| Male (N = 1238) | Ref | -0.74 (-2.48, 0.99) | 0.4001 | 0.69 (-2.46, 3.83) | 0.6688 |  |

^a^ Adjusted for age, race, education level, BMI, hypertension, diabetes, cigarette smoking, firearm noise exposure, occupational noise exposure and recreational noise exposure, OSA.

Table S3 Adjusted^a^ associations between sleep duration and PTA hearing thresholds stratified by race (N = 2777)

| Race | Normal-sleep | Short-sleep | | Long-sleep | | *P*_interaction_ |
| --- | --- | --- | --- | --- | --- | --- |
|  |  | β (95% CI) | *P* | β (95% CI) | *P* |  |
| **Low-frequency PTA** |  |  |  |  |  | 0.9599 |
| Mexican American (N = 499) | Ref | 0.20 (-1.13, 1.53) | 0.7699 | 1.00 (-0.84, 2.83) | 0.2875 |  |
| Non-Hispanic White (N = 847) | Ref | 0.73 (-0.56, 2.01) | 0.2675 | 0.66 (-1.17, 2.49) | 0.4807 |  |
| Non-Hispanic Black (N = 560) | Ref | 0.51 (-0.67, 1.69) | 0.3960 | 0.28 (-1.45, 2.02) | 0.7483 |  |
| Other races (N = 871) | Ref | 0.09 (-0.95, 1.13) | 0.8637 | 0.22 (-1.20, 1.63) | 0.7633 |  |
| **Speech-frequency PTA** |  |  |  |  |  | 0.7260 |
| Mexican American (N = 499) | Ref | 0.26 (-1.05, 1.57) | 0.6979 | 0.72 (-1.08, 2.52) | 0.4332 |  |
| Non-Hispanic White (N = 847) | Ref | 0.64 (-0.58, 1.85) | 0.3059 | 1.09 (-0.65, 2.83) | 0.2203 |  |
| Non-Hispanic Black (N = 560) | Ref | -0.25 (-1.36, 0.86) | 0.6608 | 0.34 (-1.30, 1.98) | 0.6848 |  |
| Other races (N = 871) | Ref | -0.33 (-1.31, 0.64) | 0.5024 | 0.04 (-1.29, 1.36) | 0.9587 |  |
| **High-frequency PTA** |  |  |  |  |  | 0.8488 |
| Mexican American (N = 499) | Ref | 0.61 (-1.64, 2.86) | 0.5967 | 2.59 (-0.52, 5.69) | 0.1030 |  |
| Non-Hispanic White (N = 847) | Ref | 0.02 (-2.37, 2.41) | 0.9863 | 2.81 (-0.61, 6.23) | 0.1074 |  |
| Non-Hispanic Black (N = 560) | Ref | 0.70 (-1.01, 2.42) | 0.4230 | -0.07 (-2.60, 2.45) | 0.9553 |  |
| Other races (N = 871) | Ref | -1.78 (-3.51, -0.05) | **0.0443** | 0.62 (-1.73, 2.97) | 0.6053 |  |

^a^ Adjusted for age, gender, education level, BMI, hypertension, diabetes, cigarette smoking, firearm noise exposure, occupational noise exposure and recreational noise exposure, OSA.
